# High frequency of chlamydial co-infections in clinically healthy sheep flocks

**DOI:** 10.1186/1746-6148-7-29

**Published:** 2011-06-16

**Authors:** Hannah Lenzko, Udo Moog, Klaus Henning, Robert Lederbach, Roland Diller, Christian Menge, Konrad Sachse, Lisa D Sprague

**Affiliations:** 1 Institut für Molekulare Pathogenese, Friedrich-Loeffler-Institut, Naumburger Straße 96a, 07743 Jena, Germany; 2 Tiergesundheitsdienst, Thüringer Tierseuchenkasse, Victor-Goerttler-Straße 4, 07745 Jena, Germany; 3 Institut für Epidemiologie, Friedrich-Loeffler-Institut, Seestraße 55, 16868 Wusterhausen, Germany; 4 Landesamt für Lebensmittelsicherheit und Verbraucherschutz, Abteilung Veterinäruntersuchung, Tennstedter Straße 8/9, 99947 Bad Langensalza, Germany; 5 Institut für Bakterielle Infektionen und Zoonosen, Friedrich-Loeffler-Institut, Naumburger Straße 96a, 07743 Jena, Germany

## Abstract

**Background:**

The epidemiological situation of ovine chlamydial infections in continental Europe, especially Germany is poorly characterised. Using the German state of Thuringia as a model example, the chlamydial sero- and antigen prevalence was estimated in thirty-two randomly selected sheep flocks with an average abortion rate lower than 1%. Seven vaccinated flocks were reviewed separately.

**Results:**

A wide range of samples from 32 flocks were examined. Assumption of a seroprevalence of 10% (CI 95%) at flock level, revealed that 94% of the tested flocks were serologically positive with ongoing infection (i.e. animals with seroconversion) in nearly half (47%) of the flocks. On the basis of an estimated 25% antigen prevalence (CI 95%), PCR and DNA microarray testing, together with sequencing revealed the presence of chlamydiae in 78% of the flocks. The species most frequently found was *Chlamydophila (C*.) *abortus *(50%) followed by *C. pecorum *(47%) and *C. psittaci *genotype A (25%). Mixed infections occurred in 25% of the tested flocks. Samples obtained from the vaccinated flocks revealed the presence of *C. abortus *field samples in 4/7 flocks. *C. pecorum *was isolated from 2/7 flocks and the presence of seroconversion was determined in 3/7 flocks.

**Conclusions:**

The results imply that chlamydial infections occur frequently in German sheep flocks, even in the absence of elevated abortion rates. The fact that *C. pecorum *and the potentially zoonotic *C. psittaci *were found alongside the classical abortifacient agent *C. abortus*, raise questions about the significance of this reservoir for animal and human health and underline the necessity for regular monitoring. Further studies are needed to identify the possible role of *C. psittaci *infections in sheep.

## Background

Two species of the genus *Chlamydophila (C.) *have been reported to cause infections in sheep, *C.abortus*, and *C. pecorum*. Enzootic abortion of ewes (EAE) or ovine enzootic abortion, induced by *C. abortus *is particularly feared, not only because of the major financial losses it can produce in the affected flocks [1] but also due to its zoonotic potential. Exposure of pregnant women to infected sheep can lead to severe septicaemia in the mother resulting in spontaneous abortion or stillbirth of the child [2,3]. Ewes are usually infected via the oro-pharyngeal or respiratory route. Infected animals generally show no signs of disease until the following pregnancy, when chlamydiae invade and colonise the placental tissues [4,5]. EAE is a notifiable disease in Germany and also notifiable to the OIE.

Occasional cases of aborted lambs caused by *C. pecorum *have also been reported [6]. In susceptible animals, especially lambs, infection can induce, according to the subtype, pneumonia, polyarthritis, conjunctivitis, and enteritis [6]. Infection occurs through inhalation, ingestion, and direct contact. Rodolakis et al. [7] mention the occurrence of clinically inapparent *C. pecorum *infections in both abortion affected and non-affected flocks of small ruminants. The agent is not known to cause disease in humans [8].

There are very few detailed epidemiological data regarding the distribution of ovine chlamydial infections in countries outside the UK. Most of the few existing German studies are outdated and based on materials submitted for routine laboratory examination. Moreover, virtually all data have been obtained from serological studies, which are unable to discriminate among *Chlamydiaceae *spp. and interpretation of the results is cumbersome due to the variety of applied techniques [9-12]. Accordingly, the aim of this study was to estimate the chlamydial sero- and antigen prevalence in sheep flocks using the state of Thuringia as a model example.

## Methods

### Sheep flocks and sampling procedures

Flocks with an average abortion rate lower than 1% and distributed throughout the state of Thuringia were chosen at random (Table 1). The flocks had to contain more than 100 ewes, and not been vaccinated against chlamydia in the past ten years. Flocks were kept on pasture, lambing took place in-doors. The sample size was calculated as such that an estimated seroprevalence of 10% and an estimated antigen prevalence of 25% at flock-level would be detected with 95% confidence [13].

**Table 1:** Summary of the sampled flocks and collected samples

				# of samples taken						
				
flock #	flock size (ewes)	vaccine Status	abortion rate (%)^§^	1^st ^serum d1/2 **p.p**.	2^nd^ serum d21 **p.p**.	vaginal swab/ PCR	vaginal swab/ culture	rectal swab	afterbirth	foetus/foetal swab
1	500	-	0	-	29	11	11	11	-	-
2	1000	-	0	29	25	11	11	11	9	-
3	1800	-	< 1	29	27	11	11	11	12	4
4	1300	-	0	29	29	11	11	11	-	-
5	1200	-	3	29	6	11	11	11	3	4
6	500	-	0	29	27	11	11	11	-	-
7	450	-	0	29	25	11	11	11	9	-
8	1100	-	< 1	30	30	11	11	11	-	-
9	1800	-	0	29	29	11	11	11	-	-
10	550	-	0	29	29	11	11	11	3	-
11	700	-	< 1	29	29	11	11	11	-	-
12	330	-	0	30	29	11	11	11	2	-
13*	2500	+	2	30	30	11	11	11	11	-
14	400	-	< 1	29	28	11	11	11	-	-
15	700	-	< 1	29	28	11	11	11	-	-
16	750	-	< 1	29	29	11	11	11	-	-
17	900	-	< 1	29	29	11	11	11	3	-
18	250	-	< 1	29	29	11	11	11	-	-
19*	450	+	< 1	29	27	11	11	11	1	-
20	1000	-	< 1	29	25	11	11	11	-	-
21	500	-	< 1	29	29	11	11	11	-	-
22	406	-	< 1	29	29	11	11	11	-	-
23	700	-	< 1	29	29	11	11	11	-	-
24	700	-	0	29	19	11	11	11	-	-
25	550	-	< 1	29	25	11	11	11	-	-
26	1000	-	1	29	29	11	11	11	-	-
27	317	-	< 1	29	29	11	11	11	-	-
28*	350	+	6	29	29	11	11	11	1	2
29	420	-	0	29	27	11	11	11	-	-
30*	1178	+	< 1	29	25	11	11	11	-	-
31*	500	+	< 1	29	29	11	11	11	-	-
32	120	-	0	29	29	11	11	11	4	-
33	115	-	< 1	29	28	11	11	11	11	-
34	400	-	< 1	29	29	11	11	11	1	-
35	650	-	< 1	29	29	11	11	11	-	-
36*	133	+	0	29	28	11	11	11	-	-
37	377	-	< 1	29	26	11	11	11	2	-
38*	409	+	< 1	29	28	11	11	11	-	-
39**	25	-	0	25	24	11	11	11		
40	460	-	< 1	29	-	11	11	11	-	-

**total**				**1130**	**1059**	**440**	**440**	**440**	**72**	**10**

^§^according to the sheep farmers and the Thuringian sheep health authority

*due to a vaccination history not included in the prevalence study but analysed separately

**excluded from the prevalence study due to flock size

Sera and swabs were collected during the lambing seasons by and under the supervision of a veterinarian (UM) from the Thuringian sheep health service during his flock management visits between February 2009 to June 2009 (flocks 1-13) and December 2009 to April 2010 (flocks 14-40). In practice, the sampling was carried out as follows: Paired serum samples were collected from 29 ewes per flock on day 1 or day 2 post partum and three weeks later. Additionally, two vaginal and one rectal swab were obtained from 11/29 ewes. Afterbirths and foetuses occurring in the flocks were collected when available. A total of 2189 serum samples, 880 vaginal swabs, 440 rectal swabs, 72 afterbirths and 10 foetuses/foetal/pharyngeal swabs were collected (Table 1). Due to the later discovery of a vaccination history, seven flocks (13, 19, 28, 30, 31, 36, and 38) were excluded from the prevalence study and analysed separately. An eighth flock (39) was excluded due to the small flock size. For the prevalence study a total of 1740 serum samples, 704 vaginal swabs, 352 rectal swabs, 59 afterbirths, and 8 foetuses/foetal/pharyngeal swabs from 32 flocks were evaluated.

### Sample preparation and conservation

Blood was drawn from the jugular vein with a 14 gauge needle into 7.5 mL serum Monovettes (Kabe GmbH, Nümbrecht-Elsenroth, Germany) and stored upright at RT for 12 h. The Monovettes were then centrifuged at 1500 × *g *for 10 min, the supernatant serum removed and stored at -20°C until further use. Vaginal swabs (Nerbe plus GmbH, Winsen/Luhe, Germany) collected for subsequent cell culture cultivation were transferred to 1 mL of sucrose phosphate glutamate buffer (SPG, 218 mM sucrose, 38 mM KH_2_PO_4_, 7 mM K_2_HPO_4_, 5 mM L-glutamic acid) supplemented with 0.1% (w/v) bovine serum albumin V, and stored at -80°C. Vaginal swabs collected for nucleic acid extraction were transferred to 200 μl lysis buffer [(6 M guanidiumisothiocyanate, 10 mM urea, 20% (v/v) Triton X-100 and 10 mM Tris HCl (pH 4.4); Roche Diagnostics, Mannheim, Germany)] and stored at 4°C. Afterbirths and organ samples from aborted foetuses were stored at -80°C until further use.

### ELISA

Serum samples were tested for the presence of chlamydial antibodies using the CHECKIT™ Chlamydia Test Kit (Idexx GmbH, Ludwigsburg, Germany) according to the manufacturer's instructions. All measurements were performed in duplicate and matching serum pairs were analysed on the same microtitre plate. Results were normalised using the positive and negative control sera provided in the kit and expressed as percentage of the positive control according to the following formula: [(OD sample - OD negative control)/(OD positive control - OD negative control)] × 100. Sera with values below 30% were considered negative, sera with values between 30 and 40% were considered inconclusive, and sera with values greater 40% were considered as positive. Animals were considered to have seroconverted when a change from negative to positive between the first and second serum sampling was observed.

### Cell culture and isolation of chlamydiae

Buffalo Green Monkey (BGM) cells in Minimal Essential Medium (MEM) supplemented with 5% foetal calf serum were seeded into trac bottles containing glass cover slips (Dunn Labortechnik GmbH, Asbach, Germany) and maintained in a humidified atmosphere with 5% CO_2 _at 37°C. The cell monolayers were assessed for confluent growth on the day of inoculation.

Vaginal swabs were ultrasonicated (ten 6 s impulses at an amplitude of 80%; Branson 450D digital sonifier), and between 30 and 300 μL of the solution used for inoculation of five trac bottles per sample. Organ samples were thawed, mechanically disrupted, resuspended in 3 mL PBS, and ultrasonicated (two 30 s impulses at an amplitude of 80%). The resulting suspension was centrifuged for 15 min at 500 × *g *and the supernatant used for inoculation of five trac bottles containing Nephros LP medium (BioWhittacker, Walkersville, USA) with nystatin (25 E/mL), 0.4% (v/v) gentamicin (40 μg/mL), and 0.1% (v/v) vancomycin-HCl (25 μg/mL). Upon inoculation, the trac bottles were centrifuged for 60 min at 3,400 × *g *and 37°C and subsequently incubated for 2 h. After replacement of medium, the cells were incubated for a further 18 h after which medium was again renewed. Between two to ten days after inoculation, one cover slip was fixed in methanol, and the monolayer was stained for 15 min at 37°C with 25 μL of the test reagent provided in the IMAGEN™ Chlamydia kit (Oxoid Ltd., Cambridge, UK). This kit uses a *Chlamydiaceae*-specific monoclonal fluorescent antibody recognising surface LPS from all known chlamydial species. Specimens were considered positive if they showed inclusions of typical chlamydial morphology in the form of bright apple-green, smooth-edged spots. If no specific fluorescent reaction was observed after two passages, specimens were considered negative.

### DNA extraction from samples

DNA from vaginal, rectal, foetal, and foetal organs swabs as well as from pharyngeal swabs of premature born live lambs was isolated using the High Pure PCR Template Preparation Kit™ (Roche Diagnostics) according to the manufacturer's instructions. Organ samples were cut into 50 mg sections, mechanically disrupted and digested over night in 200 μL lysis buffer with 40 μL proteinase K (20 mg/mL) (Roche Diagnostics) at 37°C.

### Polymerase chain reaction (PCR)

#### Real-time PCR

Real-time PCR assays for *Chlamydiaceae, C. abortus, C. pecorum*, and *C. psittaci *were conducted as duplex amplifications in Thermo-Fast 96-well microplates (Biodeal, Markkleeberg, Germany) using a Stratagene Mx3000P Thermocycler (Agilent Technologies, Santa Clara, CF, USA) according to the method of [14]. Each reaction included an internal amplification control as described by [15], using primers EGFP1-F (GAC CAC TAC CAG CAG AAC AC), and EGFP10-R (CTT GTA CAG CTC GTC CAT GC) at a final concentration of 300 nM each, as well as TaqMan probe EGFP-HEX (HEX-AGC ACC CAG TCC GCC CTG AGC A-BHQ1) at 200 nM. Plasmid IC2 (0.5 μl, 10,000 copies) served as the internal control template (Intype IC-DNA, Labordiagnostik Leipzig, Germany). The final 25 μl reaction mixture included 12.5 μl of TaqMan GenEx Master Mix supplemented with ROX (Applied Biosystems, Darmstadt, Germany), 0.8 μM primers, and 0.2 μM probes and 1 μl of sample DNA. Cycling parameters were as follows: initial denaturation step at 95°C for 10 min, 45 cycles of 95°C for 15 s and 60°C for 60 s (annealing and extension). The cycle threshold value (Ct) was calculated by the instrument's software MxPro3000P v 4.01.

#### Nested PCR

Samples with Ct values between 38 and 40 in the *Chlamydiaceae*-specific real-time PCR were additionally analysed with a modified version of a nested PCR procedure. This PCR targets the variable domains (VD) III and IV of the *omp*A specific for all *Chlamydiaceae *[16]. Conventional PCR was carried out in a T3 Thermocycler (Biometra GmbH, Göttingen, Germany). The amplification product obtained by the outer primer pair 191CHOMP/CHOMP371 and the inner primer pair 201CHOMP/CHOMP336s was subsequently used for sequencing when species-specific assays were negative. For the outer PCR, 35 cycles were completed, each consisting of 15 s denaturation at 95°C, 30 s annealing at 50°C, and 30 s elongation at 72°C. A final elongation step of 30 s at 72°C completed the run. The same conditions also applied to the inner PCR, except that the annealing conditions were 30 s at 60°C and only 20 cycles were run. From each PCR reaction, 15 μL were analysed by agarose gel electrophoresis (2% w/v in Tris Borate EDTA buffer).

#### Biotinylation PCR

For subsequent species-specific microarray hybridization, DNA of randomly selected samples was amplified and biotin-labelled according to the method described by [17] using the primers U23F-19 and 23R-22. The temperature-time profile was 60°C/60 s for the initial denaturation, 40 cycles of 94°C/30 s, 55°C/60 s, 72°C/45 s and a final extension step at 72°C/240 s. From each PCR reaction, 10 μL were analysed by agarose gel electrophoresis (1.5% w/v in Tris Borate EDTA buffer).

DNA samples for successive *C. psittaci *genotyping were amplified and biotinylated by means of a duplex PCR procedure described by [18]. This PCR uses two primer pairs, VD1-f/VD2-r, and 201CHOMP/ompA-rev, which encompass all the variable domains of the *omp*A of *C. psittaci*. The following PCR cycling conditions were used: initial denaturation at 96°C/60 s, 40 cycles of 94°C/30 s, 50°C/60 s, 72°C/30 s and a final extension step at 72°C/240 s. From each PCR reaction, 10 μL were analysed by agarose gel electrophoresis (1.5% w/v in Tris Borate EDTA buffer).

### PCR-RFLP

In order to differentiate *C. abortus *field strains from the vaccine strain a PCR-Restriction Fragment Length Polymorphism (RFLP) assay was conducted as described by [19] using the primer pairs CAB153-F/CAB153-R, CAB636-F/CAB636-R and CAB648-F/CAB648-R. Due to three point mutations in the coding sequences of the vaccine strain 1B, specific restriction sites are lost thus enabling the differential identification of field strains. PCR cycling conditions were: an initial 15 min denaturation step at 95°C followed by 30 cycles 95°C/30 s, 60°C/30 s (CAB636-F/R and CAB648-F/R), 58°C/30 s (CAB153-F/R), 72°C/3 s and a final extension step at 72°C/10 min. Next, 10 μL of each PCR product was digested with 2 U of the respective enzyme, i.e. *Sf*cI for CAB153, *Hae*III for CAB636 and *Sau*3A for CAB648, at 37°C over night. Ten μL of the amplification products and restriction fragments (Table 2) were analysed by agarose gel electrophoresis (2% w/v in Tris Borate EDTA buffer).

**Table 2:** PCR-RFLP pattern of *C.abortus *vaccine strain and *C. abortus *field strains

coding sequence	restriction enzyme	fragment size vaccine (bp)	fragment size field strain (bp)
CAB153	*Sfc*I	319	177 + 142
CAB636	*Hae*III	118	74 + 44
CAB648	*Sau*3A	87 + 90	87 + 69 + 21

### Identification of chlamydiae and genotyping of *C. psittaci *using DNA microarray assays

Selected samples were examined by means of the ArrayTube™ (AT) DNA microarray assay (Alere, formerly Clondiag, Jena, Germany) as described by Borel et al. (2008) to corroborate the results obtained in the species-specific real-time PCRs. Hybridization signals were measured at room temperature with the ATR-01 array tube reader and analysed using the Iconoclust software version 2.2 (both Alere).

In addition to *omp*A sequence alignments and BLAST search, a DNA microarray genotyping assay based on the ArrayTube™ technology was used to determine the genotype of the *C. psittaci *strains according to the procedures described by [18].

### ompA Sequencing

In cases of inconclusive PCR results, i.e. positive in the *Chlamydiaceae *or nested PCR but with CT values > 38 in the species-specific PCR, samples were analysed by means of partial sequencing of the *omp*A. Amplicons were purified and concentrated using the DNA Clean & Concentrator™-5 kit (ZymoResearch, Freiburg, Germany) according to the manufacturer's instructions. Sequencing of the amplicons using the primer pair 201CHOMP/CHOMP336s was performed at Eurofins MWG Operon (Ebersberg, Germany). Species identity based on the *omp*A FASTA sequences was established following a BLAST search.

### Statistical analyses

In order to assess how the sampling point influenced the isolated species and the frequency of detection the Wilcoxon rank-sum test was used. For the analysis of how flock size correlated with finding ELISA (2^nd ^serum sample) and/or PCR positive animals the Spearman-Rho coefficient was calculated. In both tests, a p-value < 0.05 was considered to be statistically significant. All tests were conducted using the statistical package SPSS, release 12.0.1 for Windows (SPSS Inc., Chicago, IL., USA).

## Results

### Serology

A flock was considered sero-positive if at least one animal was tested positive in the ELISA. With the exception of two flocks (12, 40) all unvaccinated flocks were serologically positive (94%; CI 95). Moreover, seroconversion in the tested ewes was seen in 15/32 (47%) flocks (Table 3). All vaccinated flocks were serologically positive (7/7) and seroconversion of single animals was observed in three (43%) flocks (Table 4).

**Table 3:** Summation of positive sera from the unvaccinated flocks

		# positive sera vs. # sera tested		# of animals	
		
flock #	abortion rate (%)	1^st ^serum **d1/2 p.p**.	2^nd ^serum **d21 p.p**.	positive/ # total	sero-conversion/ # total
1	0	0	4/29	4/29	-
2	0	5/29	2/25	5/29	0/25
3	< 1	6/29	5/27	6/29	0/29
4	0	11/29	10/29	11/29	0/29
5	3	11/29	4/6	13/29	**2/6**
6	0	1/29	2/27	2/29	**1/27**
7	0	6/29	6/25	9/29	**3/25**
8	< 1	6/30	5/30	6/30	0/30
9	0	7/29	12/29	14/29	**6/29**
10	0	1/29	1/29	1/29	0/29
11	< 1	3/29	5/29	5/29	**2/29**
12	0	0/30	0/29	0/30	0/29
14	< 1	1/29	2/28	2/29	**1/28**
15	< 1	9/29	9/28	9/29	0/28
16	< 1	8/29	8/29	9/29	**1/29**
17	< 1	15/29	16/29	16/29	**1/29**
18	< 1	1/29	1/29	1/29	0/29
20	< 1	8/29	6/25	8/29	0/29
21	< 1	3/29	2/29	3/29	0/29
22	< 1	8/29	9/29	10/29	**1/29**
23	< 1	7/29	7/29	8/29	**1/29**
24	0	2/29	1/19	2/29	0/19
25	< 1	5/29	4/25	5/29	0/25
26	1	6/29	6/29	6/29	0/29
27	< 1	4/29	5/29	6/29	**2/29**
29	0	3/29	2/27	3/29	0/27
32	0	1/29	2/29	2/29	**1/29**
33	< 1	2/29	2/28	2/29	0/28
34	< 1	3/29	6/29	7/29	**4/29**
35	< 1	5/29	10/29	10/29	**5/29**
37	< 1	1/29	2/26	2/29	**1/26**
40	< 1	0/29	-	0/29	-

**Table 4:** Summary of positive sera from the vaccinated flocks

	**abortion rate**^§^			**# positive sera vs**. # sera tested		# of animals	
			
flock #	prior to vaccination	upon vaccination	vaccination*	1^st^ serum **d1/2 p.p**.	2^nd ^serum **d21 p.p**.	total	sero-conversion
13	1-10%	0.8-2%	since 1995 all teggs	3/30	4/30	4/30	**1/30**
19	7%	< 1%	in 2004 complete flock; since 2005 all teggs	6/29	4/27	6/29	0/27
28	2009: 17%	6%	since 1998 all teggs; in 2009 complete flock	9/29	9/29	9/29	0/29
30	< 1%	< 1%	since 1980 all teggs	2/29	0/25	2/29	0/25
31	< 1%	< 1%	since 2002 all teggs	2/29	2/29	2/29	0/29
36	< 1%	0	since 1995 all teggs	2/29	3/28	4/29	**2/28**
38	2003: 49%	< 1%	in 2003 complete flock; since 2004 all teggs	3/29	4/28	4/29	**1/28**

*Ovilis Enzovax (Intervet Deutschland GmbH, Unterschleißheim, Germany)

^§ ^according to the sheep farmers and the Thuringian sheep health authority

### Isolation of chlamydiae

A total of 23 PCR positive vaginal swabs (flocks 1-8, 11, 14, 17, 18, 23, 26, 27, 28, 34-36, 38, 40) and three afterbirths (flocks 17, 28, 23) were analysed. *C. abortus *was isolated and propagated from three vaginal swabs taken from flocks 5 (abortion), 17 and 35 (both normal births) and from a placenta in flock 28 (abortion). The latter was a field strain as determined by PCR-RFLP. *C. pecorum *was isolated and propagated from a vaginal swab in flock 14 (normal birth). Vaginal swabs taken in flocks 3, 7, 11, 34, and 38 (normal births), initially showed signs of chlamydial growth and were positive for *C. abortus *in the species-specific real-time PCR, however, no isolates could be obtained.

### Detection of chlamydiae by means of PCR

Unvaccinated flocks were considered antigen positive if at least one animal was tested positive for chlamydial DNA (Tables 5, 6). *Chlamydiaceae*-specific real-time or conventional PCR revealed the presence of chlamydia in 25/32 flocks (78%; CI 95%). Species-specific real-time PCR detected *C. abortus *in 15/32 flocks (47%), *C. pecorum *in 13/32 flocks (41%), and *C. psittaci *in 8/32 flocks (25%). In 10/32 flocks (31%) more than one chlamydial species was found, i.e. the combination *C. abortus *and *C. psittaci *(flocks 1, 3, 4, 5, 8, 11), *C. psittaci *and *C. pecorum *(flocks 1, 2, 6) or *C. abortus *and *C. pecorum *(flocks 11, 17, 19). In three (9%) of these flocks (1, 5, 8) all three species were detected (Tables 5, 6). Simultaneous detection of more than one chlamydial species per swab occurred in 9/32 flocks (28%) (flocks 1, 2, 3, 4, 5, 8, 11, 17, and 19).

**Table 5:** Compilation of the PCR, sequencing, and microarray results from the vaginal swabs of the unvaccinated flocks

	*Chlamydiaceae*			*Chlamydiaceae *species identification							
	
	detection method [# positive vs. # tested sample]			detection method [# positive vs. # tested sample]			species detected^$^				
	
flock #	23 S RT PCR^§^	nested PCR	positive vaginal swabs	species-specific RT PCR	sequencing CHOMP	microarray	ps	pc	ab	m	
1	2/11	2/11	3/11	3/11	1/11	1/11	2	-	-	1	ab,ps
2	0/11	1/11	1/11	1/11	-	-	1	-	-	-	ps
3	1/11	1/11	2/11	2/11	-	-	1	-	-	1	ab,ps
4	0/11	1/11	1/11	0/11	1/11	-	1	-	-	-	ps
5	6/11	3/11	9/11	9/11	-	1/11	-	-	3	6	ab,ps
6	5/11	0/11	5/11	3/11	2/11	-	5	-	-	-	ps
7	1/11	3/11	4/11	2/11	4^#^/11	-	3	-	-	-	ps, su^#^
8	1/11	0/11	1/11	1/11	-	-	-	-	-	1	ab,ps
9	neg.	-	0/11	-	-	-	-	-	-	-	-
10	neg.	-	0/11	-	-	-	-	-	-	-	-
11	1/11	0/11	1/11	1/11	-	1/11	-	-	1	-	ab
12	neg.	-	0/11	-	-	-	-	-	-	-	-
14	1/11	0/11	1/11	1/11	-	1/11	-	1	-	-	pc
15	neg.	-	0/11	-	-	-	-	-	-	-	-
16	neg.	-	0/11	-	-	-	-	-	-	-	-
17	11/11	-	11/11	11/11	-	-	-	-	11	-	ab
18	0/11	1/11	1/11	0/11	1/11	-	-	-	1	-	ab
20	neg.	-	0/11	-	-	-	-	-	-	-	-
21	neg.	-	0/11	-	-	-	-	-	-	-	-
22	neg.	-	0/11	-	-	-	-	-	-	-	-
23	1/11	-	1/11	1/11	-	1/11	-	-	1	-	ab
24	neg.	-	0/11	-	-	-	-	-	-	-	-
25	neg.	-	0/11	-	-	-	-	-	-	-	-
26	0/11	2/11	2/11	1/11	1/11	-	-	-	2	-	ab
27	1/11	-	1/11	1/11	-	-	-	-	1	-	ab
29	neg.	-	0/11	-	-	-	-	-	-	-	-
32	neg.	-	0/11	-	-	-	-	-	-	-	-
33	neg.	-	0/11	-	-	-	-	-	-	-	-
34	6/11	0/11	6/11	6/11	-	-	-	-	6	-	ab
35	1/11	1/11	2/11	2/11	-	1/11	-	-	2	-	ab
37	neg.	-	0/11	-	-	-	-	-	-	-	-
40	1/11	-	1/11	1/11	-	-	-	1	-	-	pc

**^§^**0 = ct > 38; ab: **^$^***C. abortus*; ps: *C. psittaci*; pc: *C. pecorum*; su:^#^*C. suis*; m: mixed infection;

- : not determined; neg.: negative;

**Table 6:** Compilation of the PCR, sequencing, and microarray results from the rectal swabs of the unvaccinated flocks

	*Chlamydiaceae*			*Chlamydiaceae *species identification							
	
	detection method [# positive vs. # tested sample]			detection method [# positive vs. # tested sample]			species detected^$^				
	
flock #	23 S RT PCR^§^	nested PCR	positive rectal swabs	species-specific RT PCR	sequencing CHOMP	DNA microarray	ps	pc	ab	m	
1	1/11	7/11	8/11	8/11	-	1/11	1	-	-	7	ab,ps,pc
2	0/11	2/11	2/11	1/11	2/11	-	1	-	-	1	ps,pc
3	1/11	2/11	3/11	3/11	1/11	-	1	-	-	2	ab,ps
4	3/11	0/11	3/11	3/11	-	1/11	-	-	1	2	ab,ps
5	7/11	0/11	7/11	7/11	-	1/11	-	-	2	5	ab,ps,pc
6	3/11	0/11	3/11	3/11	-	-	-	3	-	-	pc
7	0/11	1/11	1/11	1/11	-	-	1	-	-	-	ps
8	1/11	1/11	1/11	1/11	-	-	-	1	-	-	pc
9	neg.	-	0/11	-	-	-	-	-	-	-	-
10	0/11	1/11	1/11	1/11	-	-	-	-	1	-	ab
11	0/11	2/11	2/11	2/11	-	-	-	-	1	1	ab,pc
12	neg.	-	0/11	-	-	-	-	-	-	-	-
14	1/11	0/11	1/11	1/11	-	-	-	1	-	-	pc
15	neg.	-	0/11	-	-	-	-	-	-	-	-
16	1/11	3/11	4/11	3/11	1/11	-	-	1	3	-	ab,pc
17	9/11	1/11	11/11	11/11	-	-	-	-	4	7	ab,pc
18	neg.	-	0/11	-	-	-	-	-	-	-	-
20	neg.	-	0/11	-	-	-	-	-	-	-	-
21	0/11	1/11	1/11	1/11	-	-	-	1	-	-	pc
22	neg.	-	0/11	-	-	-	-	-	-	-	-
23	0/11	2/11	3/11	1/11	-	-	-	-	3	-	ab
24	neg.	-	0/11	-	-	-	-	-	-	-	-
25	1/11	1/11	2/11	2/11	-	-	-	2	-	-	pc
26	neg.	-	0/11	-	-	-	-	-	-	-	-
27	neg.	-	0/11	-	-	-	-	-	-	-	-
29	1/11	0/11	1/11	1/11	-	-	-	1	-	-	pc
32	neg.	-	0/11	-	-	-	-	-	-	-	-
33	2/11	3/11	4/11	3/11	1/11	-	-	-	4	-	ab
34	0/11	1/11	1/11	1/11	-	-	-	-	1	-	ab
35	0/11	2/11	2/11	1/11	1/11	-	-	-	2	-	ab
37	1/11	0/11	1/11	1/11	-	-	-	1	-	-	pc
40	neg.	-	0/11	-	-	-	-	-	-	-	-

**^§^**0 = ct > 38; ab: **^$^***C. abortus*; ps: *C. psittaci*; pc: *C. pecorum*; m: mixed infection;

- : not determined; neg.: negative;

Of the 53 positive vaginal swabs, 28 were positive for *C. abortus*, two for *C. pecorum*, and 13 for *C. psittaci*. Nine swabs were positive for more than one chlamydial species. One swab was positive for *Chlamydia suis *as determined by *omp*A sequencing. The species distribution obtained from the 62 positive rectal swabs was 22 for *C. abortus*, 11 for *C. pecorum*, four for *C. psittaci*, and 25 for more than one chlamydial species. The frequency of detected positive *C. psittaci *samples, *C. pecorum *samples (both p ≤ 0.05) and samples with multiple chlamydial species (p ≤ 0.01) differed significantly between vaginal and rectal swabs (Tables 5, 6). In the vaccinated flocks chlamydial DNA was detected by means of species-specific real-time PCR in 4/7 (57%) flocks (19, 28, 36, 38) which on further analysis proved to be specific for *C. abortus*. Subsequent PCR-RFLP revealed all *C. abortus *isolates to be field strains (data not shown). *C. pecorum *was additionally found in flock 28 and a *C. abortus/C. pecorum *mixed infection was detected in flock 19 (Tables 7, 8).

**Table 7:** Compilation of the PCR, sequencing and microarray results from the vaginal swabs of the vaccinated flocks

	*Chlamydiaceae*			*Chlamydiaceae *species identification							
	
	detection method [# positive vs. # tested sample]			detection method [# positive vs. # tested sample]			species detected^$^				
	
flock #	23 S RT PCR^§^	nested PCR	positive rectal swabs	species-specific RT PCR	sequencing CHOMP	DNA microarray	ps	pc	ab	m	
13	neg.	-	0/11	-	-	-	-	-	-	-	-
19	1/11	0/11	1/11	1/11	-	-	-	-	-	1	ab,pc
28	3/11	1/11	4/11	4/11	-	-	-	1	3	-	ab,pc
30	neg.	-	0/11	-	-	-	-	-	-	-	-
31	neg.	-	0/11	-	-	-	-	-	-	-	-
36	neg.	-	0/11	-	-	-	-	-	-	-	-
38	neg.	-	0/11	-	-	-	-	-	-	-	-

**^§^**0 = ct > 38; ab: **^$^***C. abortus*; ps: *C. psittaci*; pc: *C. pecorum*; m: mixed infection;

- : not determined; neg.: negative;

**Table 8:** Compilation of the PCR, sequencing, and microarray results from the rectal swabs of the vaccinated flocks

	*Chlamydiaceae*			*Chlamydiaceae *species identification							
	
	detection method [# positive vs. # tested sample]			detection method [# positive vs. # tested sample]			species detected^$^				
	
flock #	23 S RT PCR^§^	nested PCR	positive vaginal swabs	species-specific RT PCR	sequencing CHOMP	DNA microarray	ps	pc	ab	m	
13	neg.	-	0/11	-	-	-	-	-	-	-	-
19	neg.	-	0/11	-	-	-	-	-	-	-	-
28	2/11	0/11	2/11	2/11	-	-	-	-	2	-	ab
30	neg.	-	0/11	-	-	-	-	-	-	-	-
31	neg.	-	0/11	-	-	-	-	-	-	-	-
36	1/11	0/11	1/11	1/11	-	1/11	-	-	1	-	ab
38	2/11	-	2/11	2/11	-	-	-	-	2	-	ab

**^§^**0 = ct > 38; ab: **^$^***C. abortus*; ps: *C. psittaci*; pc: *C. pecorum*; m: mixed infection;

- : not determined; neg.: negative;

### Detection of chlamydiae in afterbirths and foetuses/foetal samples

*C. psittaci *was detected in seven afterbirths from ewes in flocks 2, 3 and 7. *C. abortus *was found in nine afterbirths from animals in flocks 5, 17, 28 and 33 and in all ten foetal samples. One positive swab obtained from an aborted foetus in flock 3 could not be allocated to any specific chlamydial species. Two pharyngeal swabs from premature born lambs in flock 5 contained *C. psittaci *and *C. abortus *(Table 9).

**Table 9:** Chlamydial species detected in ovine afterbirths and foetus/foetal/pharyngeal swabs

*Chlamydiaceae*							*Chlamydiaceae *species identification				
	**detection method** **[# positive vs. # tested sample]**						**detection method** **[# positive vs. # tested sample]**				**detected species^$^**
	
	**23 S** **RT PCR^§^**		**nested PCR**		**afterbirths**	**foetus/** **foetal swab^&^**	**species-** **specific** **RT-PCR**		**sequencing CHOMP**	**DNA** **microarray**	
	
**flock #**	**a**	**fs**	**a**	**fs**			**a**	**fs**			

2	0/9	-	1/9	-	1/9	-	1/9	-	1/9	-	ps
3	1/12	3/4	6/12	4/4	6/12	4/4	2/12	4/4	4/12	1/4	5x ps; 1x-; 2x ab; 2x ab/ps
5	3/3	4/4	-	4/4	3/3	4/4	3/3	4/4	-	-	ab
7	0/9	-	1/9	-	1/9	-	1/9	-	-	-	ps
10	0/3	-	-	-	0/3	-	-	-	-	-	-
12	0/2	-	-	-	0/2	-	-	-	-	-	-
13*	0/11	-	-	-	0/11	-	-	-	-	-	-
17	2/3	-	1/3	-	3/3	-	2/3	-	1/3	-	ab
19*	0/1	-	-	-	0/1	-	-	-	-	-	-
28*	1/1	-	-	-	1/1	2/2	1/1	2/2	-	1/4	ab
32	0/4	-	-	-	0/4	-		-	-	-	-
33	2/11	-	-	-	2/11	-	2/11	-	-	-	ab
34	0/1	-	-	-	0/1	-		-	-	-	-
37	0/2	-	-	-	0/2	-	-	-	-	-	-

**^§^**0 = ct > 38; a: afterbirth; fs: foetal swab;

*vaccinated flock; **^$^**ab: *C. abortus*; ps: *C. psittaci*; pc: *C. pecorum*;

^&^pharyngeal swabs from premature born lambs; - : not determined;

### Identification of chlamydiae by means of a DNA microarray assay

DNA obtained from seven vaginal swabs from ewes in flocks 1, 5, 11, 14, 23, 35 and 37, as well as three rectal swabs from ewes in flocks 1, 4, and 5, was additionally analysed by means of the microarray assay. *C. abortus *was detected in nine samples (flocks 1, 11, 23, 35 and 37), *C. pecorum *was found in one sample (flock 14) (Table 5), thus corroborating the results from the species-specific PCRs. The presence of *C. abortus *in a foetal tissue from an aborted lamb in flock 3 and in a foetal sample from flock 28 was additionally confirmed via microarray testing (Table 9).

### Genotyping of C. psittaci

The *C. psittaci *DNA was extracted from four vaginal swabs, one rectal swab (abortion) and from one afterbirth obtained from ewes in flocks 1, 2, 3, 6, and 7 was analysed with the DNA microarray genotyping assay which assigned all samples to the genotype A-VS1.

### Sequencing

Partial sequencing of the *omp*A confirmed the presence of *C. psittaci *in four vaginal swabs deriving from ewes in flocks 1, 4, 6, and 7 and in 5 afterbirths from flocks 2 and 3. The presence of *C. abortus *was confirmed in two vaginal swabs and three rectal swabs taken from ewes in flocks 16, 18, 26, 33, and 35 (Table 5, 6) as well as in one afterbirth in flock 17 (Table 9).

### Correlation between flock size and finding chlamydia-positive animals

Spearman's Rho revealed a correlation between flock size and ELISA positive animals (correlation coefficient: 0.179; p ≤ 0.01) and between flock size and PCR positive animals (correlation coefficient: 0.119; p ≤ 0.05).

## Discussion

Epidemiological data regarding the distribution of ovine chlamydiosis in Germany are scarce and are based solely on materials submitted for routine laboratory examination [9-12]. Results obtained from such studies are frequently biased, since the materials are not randomly sampled. The present study, therefore, aimed at estimating the chlamydial sero- and antigen prevalence in randomly chosen unvaccinated sheep flocks in the state of Thuringia with an average abortion status lower than 1%.

Assuming a seroprevalence of 10% at flock level, we found that 94% of the tested flocks were serologically positive with ongoing infection, i.e. animals that had undergone seroconversion during the study period, in nearly half (47%) of the flocks. A study conducted by [12] in the state of Baden-Württemberg on an arbitrary panel of sera, vaginal, foetal, and placental swabs from flocks with increased abortion rates, revealed the presence of chlamydial infection in up to 91% of the flocks. Other studies estimating the seroprevalence of *C. abortus *in small ruminants found rates ranging from 4.8% in Sardinia [20], to 29% in Switzerland [21], 36% in Slovakia [22] and 47% in north eastern Turkey [23]. However, due to numerous parameters such as differences in study design and inclusion criteria (e.g. high abortion rates), flock size and management, prevalence of other abortifacient agents (e.g. *Brucellae, Salmonellae, Toxoplasma, Coxiella (C.) burnetii*) and the detection methods applied (CFT, competitive ELISA), it is virtually impossible to compare the present study's seroprevalence findings with the afore mentioned studies. Although no clinical signs were detected in our examined herds, seroconversion occurred around time of lambing. We observed seropositive animals at both sampling times, indicating chlamydial circulation within the herds resulting in the infection of naïve or seronegative animals.

On the basis of an estimated 25% antigen prevalence, PCR analyses revealed the presence of chlamydia in 78% of the flocks in the present study. A German study found chlamydial antigen prevalence in sheep to range between 50 and 71% [12] while a British study found prevalences of chlamydial infection to vary between 5-50% [24]. Other studies found prevalence rates in small ruminants to be around 11% in southern Italy [25], 7% in Anatolia [26] and 24% in Tunisia [27]. Prevalence was either determined by antigen ELISA, cultivation, conventional or multiplex PCR. All studies were carried out on samples from flocks with higher abortion rates than in our study.

The species most frequently found in our study was *C. abortus *(50%), directly followed by *C. pecorum *(47%). Remarkably, *C. psittaci *was detected in 25% of the tested flocks. Mixed infections occurred in 25% of the tested flocks. The likelihood of detecting chlamydia by using swabs appears to be independent of the sampling site, i.e. vagina or rectum. However, the frequency of individual species detected differed significantly between vaginal and rectal swab samples. Vaginal swabs were more reliable at finding *C. psittaci *whereas *C. pecorum *and samples with mixed infections were detected significantly more often in rectal swabs. We therefore recommend taking samples from both sites so that infections with neither chlamydial species are missed. The finding that no significant differences with regard to the sampling site were observed for the detection of *C. abortus *is noteworthy and supports the possibility of intestinal infection and/or faecal-oral transmission, as already postulated by others [28,7,8]. The higher detection rate of *C. pecorum *in rectal swabs appears to reflect its assumed habitat, since sheep frequently harbour this agent in their intestine [8]. The discovery of eighteen *C. psittaci *positive swabs and seven positive afterbirths was unexpected. Only two recent reports mention the sporadic isolation of *C. psittaci *strains from sheep [29,30]. We were not able to obtain any *C. psittaci *isolates, most likely due to the low concentration of the agent in the samples as determined by real-time PCR. Other factors explaining this failure could be due to sampling technique or sample transport, low viability or amount of inocula [31-33]. Therefore, in order to confirm the real-time PCR results and to determine the genotype, we additionally analysed these samples by means of sequencing and DNA microarray. We exclude cross-contamination during sampling, as flocks were sampled on different days and in different parts of Thuringia. Moreover, in order to minimise carry over between the samples from different flocks during preparation in the laboratory, collected samples were processed and analysed flock-wise. Finally, real-time PCR revealed not only different species but also varying Ct values for the respective species within an analysed flock, which indicates differences in chlamydial loads among individual animals.

The seven *C. psittaci *positive afterbirths could all be allocated to the respective ewes. One ewe in flock 2 was positive in both serum samples but PCR negative pointing towards a recent chlamydial infection. In flock 3, one ewe had a *C. psittaci *positive vaginal swab but was serologically negative, indicating an acute, possibly intrauterine infection. A second ewe in flock 3 was PCR positive for a mixed infection with *C. psittaci *and *C. abortus *but serologically negative, also indicating a possible acute infection. Of the three ewes in flock 3, one was neither serologically nor PCR positive; the remaining two were serologically negative. This also applied to the ewe in flock 7. Since no swabs had been taken from the last three animals, we cannot exclude the possibility of an acute chlamydial infection. A further possibility explaining the presence of *C. psittaci *in the afterbirths might be contamination of the lambing quarters with avian faeces, since birds, i.e. sparrows and pigeons, had free access to them. However, the detection of *C. psittaci *in vaginal and rectal swabs indicates that these infections indeed occurred in the flocks examined. It is conceivable that the agent was transmitted to the ewes through contact with the birds. Others have reported cross-infections between cattle, sheep, and kestrels [34]. In any case, the significance of *C. psittaci *in sheep in terms of their virulence and possible zoonotic impact is still unclear and more studies are needed for clarification.

We were intrigued to find one flock (12) to be presumably free of chlamydial infection, as none of the tested animals was positive in the ELISA or in the PCR analyses. This comparatively small flock had a good hygiene regime, as assessed by the Thuringian sheep health authority according to the criteria defined in [35,36] and only introduced vaccinated rams into the flock. When looking at the flock size we noticed that increasing flock size indeed influenced the probability of finding chlamydia positive animals. It is obvious that competent flock management and high standards of hygiene are easier to maintain in smaller flocks than in larger ones. However, when looking at the vaccinated flocks, flock size was not the main criterion for healthy flocks. High rates of abortion in the smaller flocks prior to vaccination not only indicate poor flock management but also inadequate standards of hygiene. Moreover, vaccination might even mask shortcomings in hygiene.

We were somewhat surprised at the low number of seropositve animals in the vaccinated flocks and the ongoing infection in three of the flocks. A Swiss study reports the unexpectedly low rate of seroconversion after vaccination and attribute it to individual immunoreactions of the sheep [37]. The identification of wild type *C. abortus *isolates in vaccinated flocks has also been described by others [38]. Although vaccination could not eliminate *C. abortus *from the flocks, it did reduce the rate of abortion considerably, especially in flocks 28 and 38. Vaccination in combination with good flock management and hygiene appear to be a feasible approach to contain chlamydial infection within a flock [39].

Our study has shown that serological testing is an adequate technique for monitoring sheep flocks for the presence of chlamydia. However, in order to obtain a realistic picture on the distribution of the various species present in a flock, serology is not sufficient. The ELISA technique which was used in our study, for example, can not discriminate between *C. abortus, C. pecorum*, and *C. psittaci *[40,41]. Standardised or harmonised and commercially available testing systems for serology capable of differentiating *C. abortus, C. pecorum*, and *C. psittaci *would considerably improve the informative value of prevalence studies. Though sampling for DNA analysis is tedious, it does provide additional information with regard to species distribution.

## Conclusions

Our data show that unexpectedly high chlamydia prevalence rates exist in flocks with low abortion status and corroborate the findings by others [42,43]. This could imply that other factors, such as the virulence of the chlamydial strains, individual animal or breed related immunity, and presence of other microorganisms and parasites (e.g. salmonella, coxiella, toxoplasmas) might additionally be responsible for eliciting abortions. In order to determine these, future studies should compare clinically healthy chlamydia-positive flocks with flocks with clinically manifest chlamydial infection and flocks with reproductive disorders without chlamydial infections. Isolation and characterisation of ovine *C. psittaci *strains are required to assess their clinical impact and possible zoonotic potential.

## Abbreviations

EAE: Enzootic abortion of ewes; EDTA: Ethylenediaminetetraacetic acid; OD: Optical density; OIE: World Organization for Animal Health; RFLP: Restriction Fragment Length Polymorphism; RT: room temperature; VD: variable domain

## Authors' contributions

HL: collected and processed the samples and evaluated the data. UM: collected the samples and helped with the contacting of the sheep farmers. KH: helped with the ELISA and cultivation of the chlamydial isolates. RL: examined the foetuses and afterbirths. RD: designed the study and carried out the statistical analyses. CM: contributed to the interpretation of the data and helped with editing and revision of the manuscript; KS: contributed to the study design, obtained the funding, and evaluated the microarray, DNA sequencing, and PCR results and helped with the revision of the manuscript. LDS: contributed to the study design, evaluated the data, drafted, and wrote the manuscript. All authors read and approved the final manuscript.
